# Identification of racial disparities in breast cancer mortality: does scale matter?

**DOI:** 10.1186/1476-072X-9-35

**Published:** 2010-07-05

**Authors:** Nancy Tian, Pierre Goovaerts, F Benjamin Zhan, Jeff G Wilson

**Affiliations:** 1 Texas Center for Geographic Information Science, Department of Geography, Texas State University-San Marcos, 601 University Drive, San Marcos, Texas, 78666 USA; 2 BioMedware Inc., 3526 W Liberty, Suite 100, Ann Arbor, Michigan, 48103 USA; 3 Texas Center for Geographic Information Science, Department of Geography, Texas State University-San Marcos: 601 University Drive, San Marcos, Texas, 78666 USA; 4 School of Resource and Environmental Science, Wuhan University, Wuhan, Hubei, 430079 China; 5 Department of Chemistry and Environmental Sciences, University of Texas at Brownsville, 80 Fort Brown - MO1.114, Brownsville, Texas, 78520 USA

## Abstract

**Background:**

This paper investigates the impact of geographic scale (census tract, zip code, and county) on the detection of disparities in breast cancer mortality among three ethnic groups in Texas (period 1995-2005). Racial disparities were quantified using both relative (RR) and absolute (RD) statistics that account for the population size and correct for unreliable rates typically observed for minority groups and smaller geographic units. Results were then correlated with socio-economic status measured by the percentage of habitants living below the poverty level.

**Results:**

African-American and Hispanic women generally experience higher mortality than White non-Hispanics, and these differences are especially significant in the southeast metropolitan areas and southwest border of Texas. The proportion and location of significant racial disparities however changed depending on the type of statistic (RR versus RD) and the geographic level. The largest proportion of significant results was observed for the RD statistic and census tract data. Geographic regions with significant racial disparities for African-Americans and Hispanics frequently had a poverty rate above 10.00%.

**Conclusions:**

This study investigates both relative and absolute racial disparities in breast cancer mortality between White non-Hispanic and African-American/Hispanic women at the census tract, zip code and county levels. Analysis at the census tract level generally led to a larger proportion of geographical units experiencing significantly higher mortality rates for minority groups, although results varied depending on the use of the relative versus absolute statistics. Additional research is needed before general conclusions can be formulated regarding the choice of optimal geographic regions for the detection of racial disparities.

## Background

Breast cancer is the most common cancer among women and accounts alone for 31% of diagnosed cases in the United States [1]. The lifetime risk for women to develop breast cancer has nearly tripled in the last sixty years from 1 in 22 to 1 in 8 [2]. However, all persons are not equal when faced with this disease: different racial and socio-economic groups display striking disparities across regions with respect to late-stage diagnosis and mortality rates of breast cancer [3-5]. From 1998 to 2002, breast cancer mortality among African-American women was 50% higher than for white women: 36.0 deaths versus 24.4 deaths per 100,000 women, respectively [6]. African-Americans living in counties with more than 20% of the population below the poverty line had more than 6% higher mortality in breast cancer than those living in counties with less than 10% poverty line [7].

Geography is a critical factor that drives reported racial/ethnic and socioeconomic disparities through a large adverse impact on the level and quality of received health care in that African-Americans and Hispanics tend to live in different areas than the White non-Hispanic population. Hispanics disproportionally account for 56% of 3.8 millions Texans living in poverty in contrast to 17% African-Americans and 24% White non-Hispanics [8]. Health disparities related to race and socio-economic status are particularly important in a large state like Texas. Out of 12 million female people in Texas, 15,132 women were diagnosed with breast cancer in 2008; and 18.4 percent of them (about 2,780) were expected to die from the disease [9]. The massive burden of breast cancer caused by the disproportionate representation of African-Americans and Hispanics in Texas, as well as the prevalence of lower socioeconomic status (SES) in these minority populations, poses a great challenge to public health officials and policy makers.

The interpretation of aggregated health outcomes hampers the understanding of the source of detected racial and socioeconomic disparities in health [10,11]. Different geographic scales can lead to inconsistent results in health outcomes [12,13], which is referred to as modifiable areal unit problem (MAUP), well-known in the geography literature [14]. Ecological fallacy denotes the error associated with the interpretation of the nature of individuals solely based on the aggregate characteristics of the group that these individual belong to. Using zip code and census tract data, Geronimus and Bound [15] argued that less bias were introduced when smaller geographic units were analyzed. On the basis of a large national survey dataset, Soobader et al. [13] replicated Geronimus' study at the census tract and block group levels. Their findings supported that despite substantial difference between aggregate socioeconomic status (SES) proxies and individual SES, smaller geographic units resulted into a better estimate of individual socioeconomic status proxies. Furthermore, Krieger et al. [12] confirmed that smaller geographic units, such as census tracts and block groups, provided more consistent SES gradient patterns than zip code data.

The effects of geographic scales on health disparities are now well-recognized, especially with the increasing use of Geographic Information Systems (GIS). An extensive research has been focused on aggregation scale effects on proxies for individual socioeconomic measurements [10,12]. Although the evidence has supported that smaller geographic units introduce less biases in the quantification of social inequalities, there has been considerable debate on the topic [16]. On the other hand, most studies [17,18] still rely on national samples resulting in the masking of geographic variations in health across the United States. Few studies have been conducted to explore how geographic scales impact racial disparities in cancer.

Rate difference and rate ratio are the primary means to enumerate effect sizes across diverse social and racial groups [19]. No consensus has been reached on the use of either relative or absolute measurements to quantify health disparities. For comparisons over time or across geographic areas, populations, or indicators, disparities should however be measured in both absolute and relative terms since they can lead to contradictory conclusions when not considered together [20,21]. The absolute measure has important implications on health-policy making and resource allocation in that it reflects the strength of health difference. Although relative measures may not offer much insights if the risk in the compared groups is too low [22], they might be appealing to epidemiologist who routinely work with odds ratios.

Cancer outcomes vary by socioeconomic status and geographic locations which reflect underlying effects of living environment, lifestyle factors, and access to utilization of health care as well [23]. Socioeconomic disparities in health are inherited with the impact of geographic scales due to lack of individual socioeconomic information. However, the current surveillance systems do not routinely collect socioeconomic variables such as education, income and occupation from health and disease resources including medical records, cancer registries, and birth and death certificates [12]. Area-based approach enables the derivation of the socioeconomic status (SES) information at certain geographic level to assess the health disparities. However, different geographic scales can be used to quantify the SES based on data availability.

The unique demographic composition of geographies associated with racial disparities in Texas provides a suitable platform to investigate the impact of geographic scale on measuring racial disparities. This study investigates the effects of geographic scale on racial and socioeconomic disparities based on census tract, zip code, and county levels by detecting where breast cancer mortality is significantly higher for African-American and Hispanic women using the population-based data from 1995-2005 in Texas. The influence of socioeconomic status on the significance of racial disparities is explored as well in terms of percentage of population under the poverty level.

## Methods

### Geographic Boundary

The cartographic boundary shapefiles including census tract, zip code, and county are defined according to the US Census 2000 data. The state of Texas consists of 4,388 census tracts (CTs), 2,884 zip codes, and 254 counties based on the 2000 Census. As CTs generally include similar population sizes, they are smaller within metropolitan urban areas such as Houston, Dallas, and the Austin-San Antonio area. CTs are also designed to contain homogenous populations in terms of neighborhood socioeconomic composition [11] and form an optimal administrative geographic unit used to determine eligibility and resource allocation for diverse programs. The average population per census tract in Texas is 5,500 with standard deviation (SD) of 3,600. US Postal Service zip code is designed for mail delivery and each zip code covers 13,800 people on average in Texas (SD = 16,000). Counties are the primary administrative divisions of the state and the intermediate tier between state and local governments. Each county has 100,000 residents on average in Texas with SD equal to 338,000.

### Population Data

Female population by race and age category was derived from the 2000 Census Summary File 1 (SF1) 100-Percent Data. Race was defined as specific physical, hereditary and cultural traditions within social-political construct, while ethnicity is labeled by one of two groups:

Hispanic or Latino, and Not Hispanic or Latino. The following three races/ethnicities were considered in this study: White non-Hispanic, African-American (Non-Hispanic), and Hispanic. According to the 2000 Census in Texas, the female population for White non-Hispanics was 5,555,694; that of African-Americans was 1,244,302; and the female population of Hispanics or Latinos was 3,273,458. To calculate age-adjusted mortality rate in breast cancer, the population by race was grouped into five categories of age 1-14, 15-24, 25-44, 45-64, and 65+. Since only tabular information is available from SF1, population data by race and age was joined to geographic boundary shapefiles to allow for a spatial analysis.

### Mortality Data

Mortality data for female breast cancer were provided by the Center of Health Statistics, Texas Department of State Health Services (TDSHS). Cause of death and demographic information from Texas death certificates were collected by the Vital Statistics Unit from TDSHS. A total of 26, 910 mortality cases were reported in Texas through the period of 1995-2005. All records included information on street address, age group, year of death, race, Hispanic origin, and geographic location. Unfortunately, 3,220 cases (12%) were disqualified as they could not be geocoded owing to lack of a complete address, an incorrect address, or a post office mailing address. The matching rate is also slightly different across racial groups: it is 3-4% larger for African-American and Hispanic groups than for White non-Hispanic females (Table 1). One of the main challenges in the analysis of data from minority groups is the small population size, in particular for smaller geographical units such as census tracts. For example, Table 2 indicates that only 36% of census tracts and zip codes with Hispanic residents have reported at least one death from breast cancer during the eleven-year period. A similar proportion was observed for African-Americans. For both ethnic groups, this percentage raises to 60% at the county level. In comparison, White non-Hispanic females had a much larger percentage (67.97%-94.47%) at all scales.

**Table 1:** Completeness of geocoding results for different race/ethnic groups, 1995-2005

	White Non-Hispanics	African-Americans	Hispanics	Total
Unmatched	2,458	350	412	3,220
Matched	16,419	3,616	3,655	23,690
Total	18,877	3,966	4,067	27,180
Percent Matched	86.98%	91.17%	89.87%	88.03%

**Table 2:** Number of geographic units (census tract, zip code, and county) with non-zero population and mortality data by race.

Geographic Level		White Non-Hispanics	Race African-Americans	Hispanics
Census Tract (4,388)	Population	4,382	4,322	4,375
	Mortality	3,518	1,341	1,592
	Percentage	80.28%	31.03%	36.39%
Zip Code (2,884)	Population	1,889	1,620	1,866
	Mortality	1,284	597	674
	Percentage	67.97%	36.85%	36.12%
County (254)	Population	253	209	253
	Mortality	239	126	155
	Percentage	94.47%	60.29%	61.26%

The ratio of these two numbers, referred to as percentage, indicates the much smaller proportion of units where at least one death was observed for minority populations.

The case records were processed for White non-Hispanics, African-Americans, and Hispanics separately to examine the age-specific mortality rate in female breast cancer. In order to examine the mortality rate by age, the 2000 Census population by age was adopted to calculate the 18 age specific mortality rates including 0-4, 5-9, 10-14, 15-19, 20-24, 25-29,30-34, 35-39, 40-44, 45-49, 50-54, 55-59, 60-64, 65-69, 70-74, 75-79, 80-84, and 85+. Age-specific mortality rates also provide a solid ground to group cases into a larger age group in order to evaluate the mortality rate across race at the three different geographic scales. The mortality cases were grouped according to different classes of age (1-14, 15-24, 25-44, 45-64, and 65+) to compute age-adjusted mortality rates at census tract, zip code, and county levels. The five age groups were selected in order to reduce small number issues resulting from partitioning data by age and race at different geographic scales. The US 2000 Standard Million population was used to take into account age heterogeneity among populations.

### Socioeconomic Data

The paradoxical effects of socioeconomic status (SES) complicatedly impacts breast cancer incidence and mortality across races [7]. Lower SES has been reported to have a protective effect against the development of the disease [24], while it is often shown as a risk factor for breast cancer mortality [25]. The poverty rate, which is defined as the percentage of individuals living below the federal poverty threshold, is used here as a proxy index of socioeconomic status for individual cases. Poverty rate is the most important component in a composite socioeconomic index and is strongly correlated with the following socioeconomic variables [25]: percentage of population with at least high school education (-0.73), median family income (-0.77), unemployment (0.78). The current population survey from the US Census Bureau shows that 16.4% of the population in Texas (approximately 3.8 million) are living below the poverty threshold, which corresponds to an annual income of $20,614 for a family of four. The 2000 Census Summary File 3 was used to derive the poverty rate at the levels of census tract, zip code and county. At each level, data were coded into three groups: high SES (0.00-9.99%), middle SES (10.00-19.99%), and low SES (20.00% +) (Table 3). Moving up from census tract to county levels, the proportion of geographic units with middle SES increased from 33% to 69%, while this proportion decreased at both ends: 37% to 9% for low SES and 30% to 22% for high SES.

**Table 3:** Frequency of different classes of poverty levels at the census tract, zip code, and county levels.

Poverty Level		High SES (0-9.99%)	Middle SES (10.00-19.99%)	Low SES (≥20.00%)
Census Tract (4338)	N	1613	1445	1330
	Percentage	36.76	32.93	30.31
Zip Code (2884)	N	1045	1181	658
	Percentage	36.23	40.95	22.82
County (254)	N	22	175	57
	Percentage	8.66	68.90	22.44

Percentage was derived by the number of geographic units for each SES level divided by the total number of units at each geographic level.

### Methods

The measurement of health disparities has been the topic of much conceptual and pragmatic discussion [19,26]. Relative and absolute differences in health outcomes are the primary tools used to quantify changes across diverse social and racial groups. A relative measure compares the rate differences against a reference point. An absolute measure provides a simple arithmetic difference between a target group and a reference group. The most favorable group is commonly used as a reference point which all groups aim to achieve. According to a report from the Surveillance Research Program (SRP) and the Applied Research Program (ARP) of the Division of Cancer Control and Population Sciences of the National Cancer Institute [20], pairwise absolute and relative comparisons for cancer data suffice for the comparison of specific groups conducted in the present study. Relative and absolute measures provide fundamentally different information with the possibility of arriving at different conclusions. Relative statistics cannot reflect the variation patterns in health provided by absolute measures, yet they allow one to account for spatial changes in the magnitude of rates across the study area. Thus, this study adopted both absolute and relative measurements.

Accounting for population size in the computation of the disparity statistics leads to allocate greater weight to racial or socioeconomic groups with larger population. In addition, a population-weighted scheme corrects for the small number problem that is often observed for minority groups and smaller geographic units like census tracts. Fleiss [27] and Lachin [28] proposed four different population weighted statistics in absolute scales and two different ones in relative scales. Goovaerts et al. [21] assessed the six statistics through a simulation approach mimicking different scenarios in terms of the magnitude and frequency of disparities. They identified two test statistics (Equations 1 and 3 below) that had higher power and created fewer false positives using prostate and lung cancer mortality datasets for the period of 1970-1994 in the Southeastern region of the United States. The power measures the probability of correctly detecting significant disparities, while a false positive corresponds to the situation where a racial disparity is wrongly declared significant. The statistic to measure absolute differences between two racial groups is as follows:(1)

Where is  the population-weighted average of cancer rates computed as:(2)

The statistic to measure relative differences between two racial groups is as follows:(3)

In the above expressions, *p*_1_(*u_i_*) and *r*_1_(*u_i_*) denote the population size and mortality rate of the reference group in region *u_i _*(i.e. White non-Hispanic population), while *p*_2_(*u_i_*) and *r*_2_(*u_i_*) and are the same quantities measured for the disadvantaged racial groups including African-American and Hispanic. Region *u_i _*represents any geographic unit at the census tract, zip code, and county levels in Texas. The two statistics in equations (1) and (3) follow a standard normal distribution.

The null and alternative hypotheses for testing whether the difference in health outcomes between two racial groups is significant are as follows:

The hypotheses for rate difference(4)

The hypotheses for rate ratio(5)

The significance (*p*) of the above two test statistics can be assessed by comparing the test statistic against its expected distribution under the null hypothesis of equality of rates. However, hundreds of individual tests might need to be conducted, particularly when the small geographic units are analyzed such as census tracts. Consequently, false significance tests are likely to be reported without control of multiple testing. Correction for multiple testing is thus essential to avoid overestimating the proportion of significant disparities (i.e. high rate of false positives). The most widely used technique of Bonferroni method tends to be more conservative and truly significant difference will be largely unidentified [29]. The false discovery rate (FDR) approach, which controls the expected proportion of true null hypotheses out of the total number of rejections, is implemented here because it is less restrictive and more powerful than other correction methods [30].

A two-tailed test was performed with a critical α level equal to 0.05 and FDR correction using the Space-Time Intelligence System [31]. White non-Hispanic population was used as the reference population, which means that a positive rate difference (RD) indicates higher mortality for African-American and Hispanic women. Similarly, if rate ratio (RR) exceeds 1, the minority women experience significantly higher mortality rates than their White non-Hispanic counterpart. This study reports all geographic units where absolute and relative racial disparities in breast cancer mortality tested significant. The statistic was not computed and labeled as "no cases" in Figure legends whenever the number of population is zero for one of the two ethnic groups. The geographic units are not considered as well when the number of cases is zero for both ethnic groups when measured in RD and for either ethic groups when measured in RR.

## Results and Discussion

### Age-specific mortality rates

**Figure 1:**
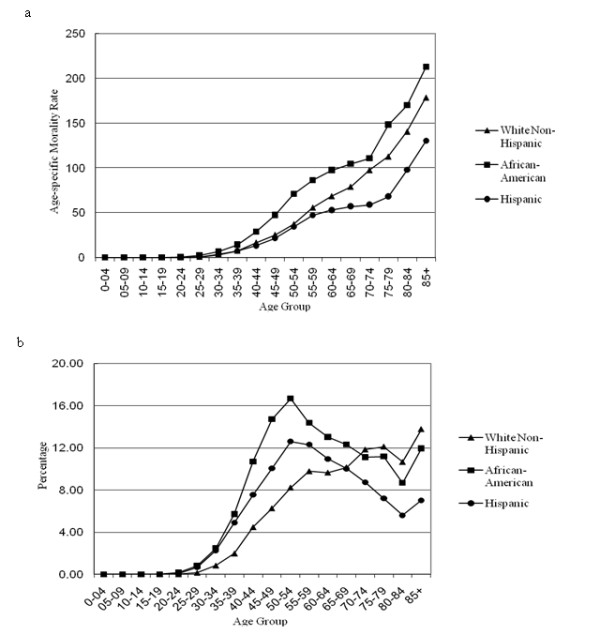
**Age-specific mortality rate (a) and percentage of mortality cases (b) for breast cancer by race in Texas, 1995-2005**.

Until their mid twenties, women of each race have close to zero mortality rates. Then, breast cancer mortality increases with age for all three racial groups (Fig. 1a). Mortality curves however never overlap and display similar ranking across ages: African-Americans have the highest mortality while Hispanics have the lowest, with intermediate rates for White non-Hispanics. There is a stronger slope to the curve for African-Americans around age 70-74, with a lag of about five years for White non-Hispanics and Hispanics. The proportion of mortality cases by age and race is shown in Fig. 1b. For African-American and Hispanic women, the mortality peaked five years earlier than White non-Hispanic females who approached their mortality crest at age 55-60.

### Racial disparities for African-Americans

**Figure 2:**
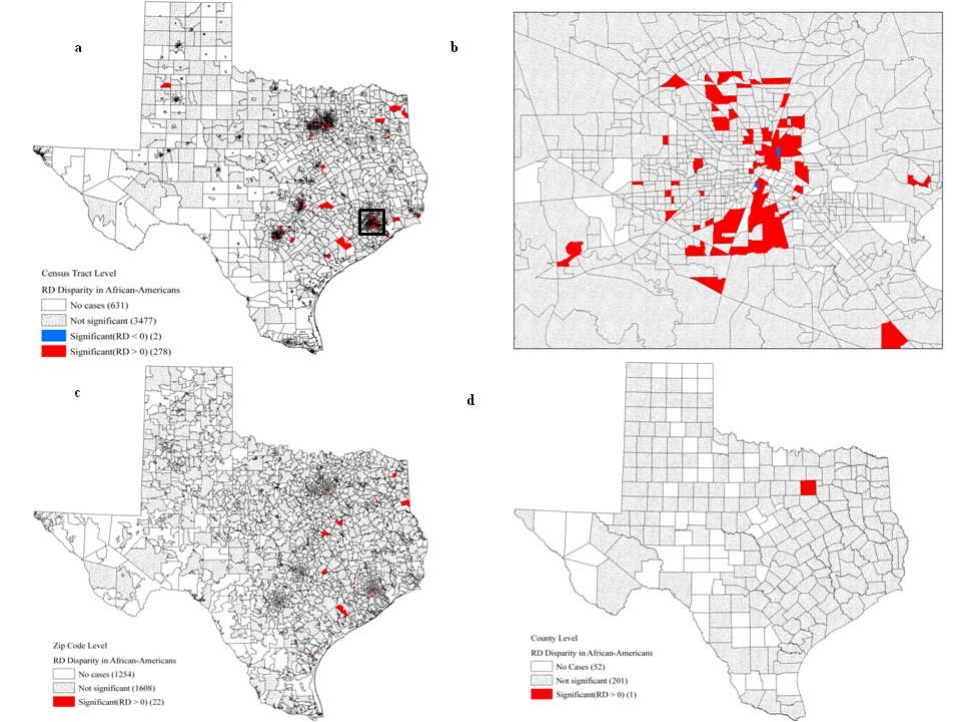
**African-American breast cancer mortality: significant racial disparities according to the RD statistic at the census tract (a,b), zip code (c), and county levels (d)**. Map b shows a magnified portion of map a (rectangle).

Table 4 lists the number of geographic units with significant racial disparities for African-American women. At the census tract level, 278 out of the total 4,388 census tracts (6.30%) displayed significantly higher mortality for African-American women compared to the White non-Hispanics in terms of rate difference (RD) measurement (Fig. 2a). Among the census tracts with significantly higher mortality for African-American women, a large proportion (87.77%) had a poverty rate greater than 10.00%. Most of the significant disparities for African-American women were found in the Houston, Dallas, and Austin-San Antonio metropolitan areas. Only two census tracts located within the Houston metropolitan areas displayed significantly higher mortality rates for White non-Hispanic women (Fig. 2b). The two census tracts had either 1 or 2 deaths for a population of 12 White non-Hispanic females in the past eleven years, which contributed to the exceptionally high mortality rates. These two census tracts had a poverty rate greater than 20%. No census tract tested for significant relative racial disparities (RR).

**Table 4:** Number of geographical units at various levels of aggregation with significant disparities in breast cancer mortality for African-American women.

Poverty Level		High SES (0-9.99%)	Middle SES (10.00-19.99%)	Low SES (≥20.00%)	Total
Census Tract (4388)	RD^1 ^< 0	0	0	2	2
	RD > 0	34	54	190	278
	RD^2 ^< 1	0	0	0	0
	RR > 1	0	0	0	0
Zip Code (2884)	RD < 0	0	0	0	0
	RD > 0	2	8	12	22
	RR < 1	0	0	0	0
	RR > 1	3	0	0	3
County (254)	RD < 0	0	0	0	0
	RD > 0	0	1	0	1
	RR < 1	0	0	0	0
	RR > 1	0	2	0	2

Units are classified according to their socioeconomic status (SES) as measured by the percentage of population below the poverty threshold.

^1^The rate difference (RD) measurement is an absolute measure of breast cancer mortality rates for Black women compared to the Whites.

^2^The rate ratio (RR) measurement is a relative measure of breast cancer mortality rates for Black women compared to the Whites.

**Figure 3:**
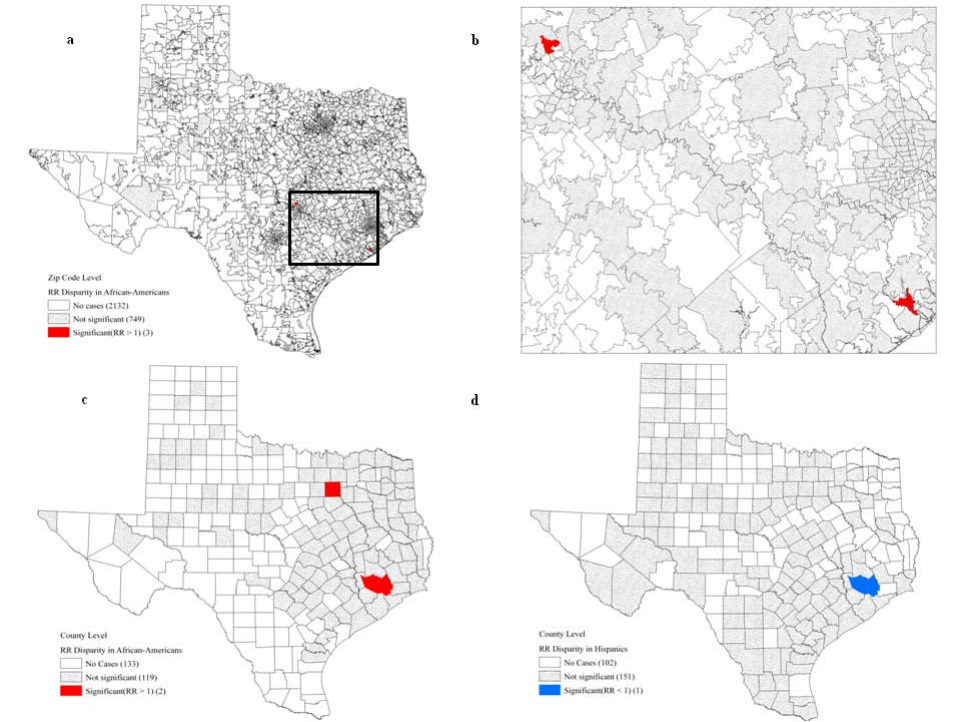
**Significant racial disparities for breast cancer according to the RR statistic**. Map a shows results for African-American at the zip code level, with a magnified portion in map b. Maps c and d display the significant disparities for African-Americans and Hispanics at the county level.

At the zip code level, no significantly higher rates, either measured in absolute or relative terms, were detected for White non-Hispanic women (Table 4). Twenty-two geographic units (0.80% of total zip code units) had significantly higher mortality for African-American in terms of RD (Fig. 2c) and three other geographic units were significant in terms of RR (Fig. 3a). Twelve out of the 22 zip codes (90.91%) located in the Southeast of Texas had a poverty level higher than 20%. The three zip codes with significant racial disparities for African-Americans were located at Cedar Park and Lake Jackson (Fig. 3b). However, their poverty rate was lower than 10.00%, which is not in agreement with the results obtained for the RD measurement.

**Figure 4:**
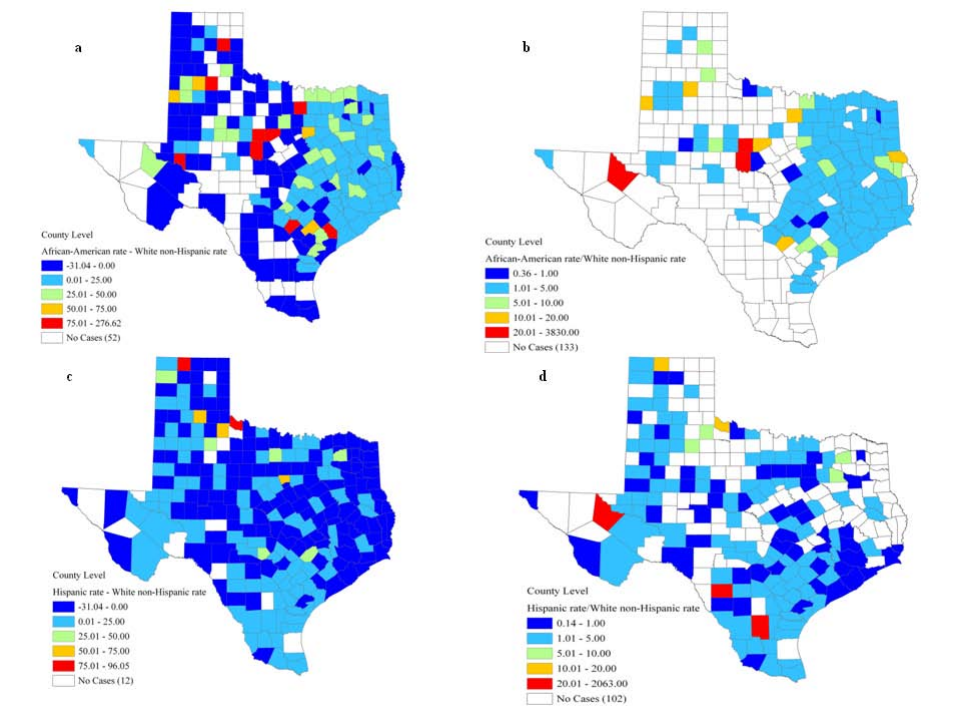
**Maps of rate difference and rate ratio between African-American (a,b) or Hispanic women (c,d) and White non-Hispanic women**.

To illustrate the magnitude and locations of racial disparities for African-American women at the county level, the difference and ratio of county-level mortality rates using White non-Hispanic women as reference were mapped in Fig. 4a and 4b. Rate difference ranged from-31.04 to 276.62 with 115 counties having higher breast cancer mortality for African-American women, while rate ratio varied from 0.36 to 3830.00 with 114 counties with a ratio larger than 1.0. However, only Dallas County (0.39% out of total counties) had a significantly higher mortality for African-American using the RD statistic (Fig. 2d), while the RR statistic led to the detection of two counties: Dallas and Harris (Fig. 3c). The age-adjusted mortality rates were 36.52 and 21.18 per 100,000, respectively, for African-Americans and White non-Hispanics at Dallas County, while the mortality rates were 36.23 and 26.96 for African-Americans and White non-Hispanics at Harris County. Regardless of the type of disparity statistic, all the counties that experienced higher mortality for African-Americans had a poverty rate between 10.00 and 19.99%.

### Racial disparities for Hispanics

**Figure 5:**
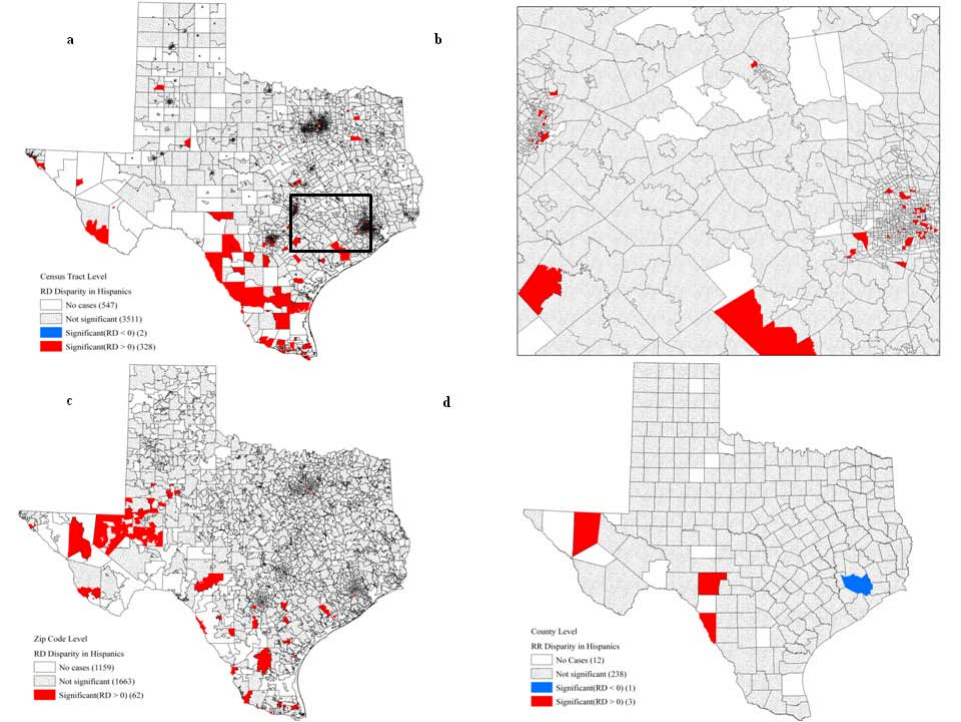
**Hispanic breast cancer mortality: significant racial disparities according to the RD statistic at the census tract (a,b), zip code (c), and county levels (d)**. Map b shows a magnified portion of map a (rectangle).

Table 5 lists, by level of geographical aggregation, the number of significant racial disparities for Hispanic women at various SES levels. Out of 4,388 census tracts, 328 had significantly higher mortality for Hispanic women (RD statistics) (Fig. 5a), while significantly higher mortality rate for White non-Hispanics were detected for only two census tracts within the Houston metropolitan area (Fig. 5b). These 328 census tracts were primarily located at the southwest border of Texas. The majority (93.90%) of census tracts with significantly higher mortality among Hispanic women had a poverty rate larger than 10.00%. However, the two census tracts with better health outcomes for Hispanic women belonged to the highest poverty level (≥ 20.00%). No significant racial disparity was found when using the RR statistic.

**Table 5:** Number of geographical units at various levels of aggregation with significant disparities in breast cancer mortality for Hispanic women.

Poverty Level		High SES (0-9.99%)	Middle SES (10.00-19.99%)	Low SES (≥20.00%)	Total
Census Tract (4388)	RD^1 ^< 0	0	0	2	2
	RD > 0	20	46	266	328
	RD^2 ^< 1	0	0	0	0
	RR > 1	0	0	0	0
Zip Code (2884)	RD < 0	0	0	0	0
	RD > 0	11	9	42	62
	RR < 1	0	0	0	0
	RR > 1	0	0	0	0
County (254)	RD < 0	0	1	0	1
	RD > 0	0	0	3	3
	RR < 1	0	1	0	1
	RR > 1	0	0	0	0

^1^The rate difference (RD) measurement is an absolute measure of breast cancer mortality rates for Hispanic women compared to the Whites.

^2^The rate ratio (RR) measurement is a relative measure of breast cancer mortality rates for Hispanic women compared to the Whites.

Units are classified according to their socioeconomic status (SES) as measured by the percentage of the population below the poverty threshold.

At the zip code level, 62 out of 2,884 (2.15%) units were identified with significantly higher mortality for Hispanic women (Fig. 5c). Eighty-two percent of these zip codes (51 out of 62) had more than 10% of their population living below the poverty line. No zip code presented significant racial disparities in terms of RR measurement. The magnitude of racial disparities between Hispanic and White non-Hispanic women fluctuated between-31.04 and 96.05 for the rate difference and from 0.14 to 2063.00 for the rate ratio. A total of 98 census tracts have higher breast cancer mortality for Hispanic women both in absolute and relative terms (Fig. 4c, d). Nevertheless, three counties in the Southwest region of Texas had significantly higher mortality rates for Hispanic women when using the RD statistic (Fig. 5d): the age-adjusted mortality rates for Hispanics were 21.03, 9.01, and 16.86 respectively for Edwards, Cuberson, and Maverick counties. The urban county of Harris had the best mortality outcome among Hispanic women for both RD (Fig. 5d) and RR (Fig 3.d): the rates were 14.97 and 26.96 respectively for Hispanics and White non-Hispanics. Moreover, 14.97 percent of population within that county lived below the poverty threshold.

## Discussion

In this study, the proportion and location of significant racial disparities changed depending on the type of statistic (absolute versus relative) and the geographic level (census tract, ZIP code, and county). The application of the RD statistic to census tract data resulted in the detection of a larger proportion of significant racial disparities than the use of the RR statistic and larger geographical units (zip code and counties). White non-Hispanics generally displayed the lower mortality rates in breast cancer. The geographic regions with significant racial disparities for African-Americans frequently have the highest poverty rate. Further, we found that Hispanic women experienced significantly higher mortality rates for breast cancer in Southwest of Texas which is characterized by a larger proportion of the population living below the poverty line.

African-American and Hispanic women experienced significantly higher mortality rates than White non-Hispanics in the southeast metropolitan areas and southwest border of Texas. More units were found significant for Hispanics than for African-Americans, which could be explained by the wider geographical distribution of Hispanic relative to African-Americans. Statistically, more differences are tested significant when measured in absolute terms (i.e. rate difference RD) than relative terms (i.e. rate ratio RR) and this proportion diminishes as the size of geographical units increases (zip code and county level). The large discrepancy between results obtained for the RD and RR statistics is caused by the measurement itself. RR statistic generally leads one to discard more geographic units because if either the reference or target group has 0 cases, the geographic units are not under consideration. Both the reference and target groups should have 0 deaths in order to exclude the geographic units in terms of RD measurement. Sometimes, absolute and relative measures can lead to different conclusions on health disparities [32]. Thus, the Centers for Disease Control recommend utilizing both absolute and relative measurement in order to fully understand the magnitude and direction of health differences, especially across geographic areas and populations [20]. Absolute disparity measurement offers important insights on making health-policy decisions and allocating health resources. Relative disparity scales may lead to miss the geographic areas with significant difference of breast cancer mortality between White non-Hispanic and other minority groups.

Racial disparities in breast cancer mortality have been well-established in ecological studies conducted at the national and state levels [33,34]. Investigating racial disparities across multiple geographic scales can help exploring the impact of using aggregation data on cancer disparities. MAUP could lead to different statistical results based on the aggregation of the same individual-level data into different spatial units [14]. For example, census tracts identified with significant racial disparities do not necessarily fall within the zip codes and counties that are declared significant. Besides, the frequency of racial disparities has increased when moving down from the county level to the census tract level, which may indicate that contextual and environmental risk factors exert different roles on health at different aggregation levels. These risk factors can include socioeconomic status, differences in treatment options, difference in cancer stage, differences in mammography screening patterns, and external environmental exposure [7,35-37]. Contrasting results on racial disparities observed at the three spatial scales may also result from the attenuation of health difference within larger geographic regions where the impact of population concentration and racial residential segregation of minorities into small and specific areas is diluted. The use of smaller geographical units allows a finer analysis of these disparities and the detection of more significant differences, as long as the minority population is large enough for a reliable estimation of rates.

Meliker and Goovaerts et al. [38] investigated racial disparities in breast and prostate cancer survival in Michigan at three different geographic levels: federal House legislative districts, state House legislative districts, and community-defined neighborhoods. The present study confirms Meliker et al's findings that analysis conducted at different scales might highlight significant racial disparities that don't overlap geographically. Meliker and Jacquez et al. [39] also concluded that the aggregation of individual-level data can lead to the detection of different clusters of breast cancer late-stage diagnosis depending on the scale of aggregation. However, unlike Meliker and Goovaerts' study [38] we found that smaller spatial supports provide more comprehensive results regarding racial disparities. Plausible explanations for such discrepancies include: 1) the use of different geographical units, which likely results into different zoning and scale effects on racial disparities, 2) the application of multiple testing correction in this study which reduced the number of significant tests, 3) the use of a different health outcome: cancer mortality instead of survival rate, 4) the larger size of minority populations that increased the reliability of rate estimates and 5) the incorporation of Hispanic ethnicity in the present study.

Changes in the geographic location of significant racial disparities across different scales seem to preclude any significant role of genetic factors on mortality rates for the racial groups studied here. Furthermore, only 5-10% breast cancer is due to the gene mutation of BRAC1 and BRAC2 [40]. Other factors, especially access to health care and screening, play an important role in determining cancer mortality. Soobader and LeClere [41] claimed that the level of geographic aggregation impacts the pathways of income inequality on individual health risk. The spatial inconsistency of racial disparity across scales may also reflect its multidimensional nature with numerous pathways taking place at different geographic levels. The choice of an appropriate scale is a critical factor to assess racial health disparities across geographic regions. Our results suggest that the census tract offers a complete and better understanding of the racial disparity in breast cancer mortality, which confirms the preferred use of income inequality at the census tract level [12]. Geronimus et al. [10] found that smaller geographic units introduced less bias when using zip code, census tract, and block groups as proxies of individual SES measurement in terms of income and education.

Significantly higher mortality rates for African-American and Hispanic women occurred in more impoverished areas of the southeast metropolitan areas and southwest border in Texas. Regions of lower socio-economic status were found to be associated with more substantial racial disparities. This association was quantitatively demonstrated by the results of a logistic regression conducted on the RD statistic at the census tract level (Table 6). Geographic units with low or middle SES have substantially higher odds ratio for African-Americans and Hispanics. A plausible explanation might be that African-Americans living in underserved areas could not access health care resources as conveniently as other races in higher SES areas. Although White non-Hispanics live in the same disadvantaged regions, these females could overcome transportation barriers, have better financial support, and access to more health care in affluent neighborhoods [42,43]. Late-stage diagnosis may happen more frequently in minorities and ultimately widens racial disparities in mortality rates within the same geographic regions [44]. On the other hand, socio-economic status is a composite statistic that mixes all races, which could be misleading in that a region dominated by minorities may have lower SES overall that does not reflect the status of White non-Hispanics in that region [4].

**Table 6:** Results of the logistic regression: odds ratios (ORs) and 95% confidence intervals (CIs) for the RD statistic at the census tract level using socioeconomic status (SES) as covariate

SES	African-American OR (95% CI)	*p*	Hispanic OR (95% CI)	*p*
High	1 (reference)	< 0.001	1 (reference)	< 0.001
Middle	1.863 (1.203 to 2.886)	0.005	2.507 (1.464 to 4.293)	0.001
Low	10.186 (7.002 to 14.818)	< 0.001	24.290 (15.296 to 38.573)	< 0.001

Although a few census tracts had significantly better mortality outcome for African-Americans and Hispanics, it is likely an artifact of the analysis caused by too small population sizes following the partitioning into such a small geographic unit (Census Tract) and different age groups. For example, the two census tracts with significantly larger mortality for White non-Hispanics within the Houston Metropolitan areas included only 12 White non-Hispanic females and 1 or 2 deaths from 1995-2005. Most of the population in the two census tracts are African-American and Hispanic women falling into the poverty class of 20.00% plus. A manifestation of the "Hispanic paradox" [45] was observed in these two tracts: Hispanic women experienced smaller breast cancer mortality (0 deaths) even though they were in a lower SES neighborhood. A similar phenomenon occurred at the county level: Hispanic women in Harris County with a poverty level above 10% had significantly lower mortality than White non-Hispanics. The racial disparities for African-American and Hispanic groups relative to White non-Hispanics provide a solid evidence of the importance of socioeconomic status in determining mortality outcomes for breast cancer. It is not surprising because impoverished neighborhoods are typically characterized by lack of sufficient health care facilities, physicians, and even appropriate cancer treatments [46].

The main limitations of the present research relate to data quality and processing. First, about 13% of White non-Hispanic cases and 10% of African-American and Hispanic cases were not geocoded correctly. This difference among ethnic groups reflects the tendency for minority to live in urban areas that have a better chance to be geocoded completely [47]. Second, age-adjusted mortality rates per race were calculated using the 2000 US census population while cases were aggregated for the period 1995-2005. In some of the geographical units, the aggregation and use of midpoint population created situations where the number of cases is larger than the population size for some of age groups. This phenomenon is more common for smaller geographical units such as census tracts. One solution to this problem is to equal the population size and number of cases for those age groups. Third, the power of the tests deserves further investigation due to small sample sizes at the census tract level. The research considering all types of cancer mortality should provide higher power to explore the scale effect on racial disparities. Fourth, the over-count and/or undercount of the population from the decennial Census may be responsible for the markedly higher mortality rates among African-American and Hispanic who tend to be undercounted more frequently [48].

## Conclusion

This study investigated both relative and absolute racial disparities in breast cancer mortality between White non-Hispanic and African-American/Hispanic women at the census tract, zip code and county levels. Analysis at the census tract level generally led to a larger proportion of geographical units experiencing significantly higher mortality rates for minority groups, although results varied depending on the use of the relative versus absolute statistics. Yet both statistics are recommended in order to fully identify significant health disparities among racial groups. A similar approach could be applied to other identifiers such as social groups. Our results are promising for health policy-makers working towards the elimination of racial disparities in cancer, in that intervention programs to modify environmental factors could be developed at smaller community-local levels. The highlighted regions with significant racial disparities could guide health-policy makers in allocating health resources more effectively. Discrepancies between our results and Meliker et al's [38] findings stress the need for additional research on the impact of geographical scale and the employment of multiple corrections on racial disparities. In particular, the analysis of different cancer outcomes including incidence, survival, mortality, and screening across geographic scales is needed before general conclusions can be formulated regarding the choice of optimal geographic regions for the detection of racial disparities. Future work should also explore how racial disparity changes across scales when adjusting for risk factors, such as socioeconomic status and access to health care (e.g. mammography screening), leading to a better understanding of differences found among racial groups. For such studies hierarchical models (i.e. multi-level modeling) would help us to evaluate how individual-level characteristics and area-based contextual factors explain differences in health outcomes across racial groups.

## Competing interests

The authors declare that they have no competing interests.

## Authors' contributions

All authors intensively participated in the study. NT and PG conceptualized the study design and the analysis of the data. FBZ and JW contributed to the results interpretation and improvement of the manuscript. The final manuscript is approved by all authors.
